# High school students’ knowledge of endangered fauna in the Brazilian Cerrado: A cross-species and spatial analysis

**DOI:** 10.1371/journal.pone.0215959

**Published:** 2019-04-25

**Authors:** Marcos Aurélio de Amorim Gomes, Tatiel Venâncio Gonçalves, Fabrício Barreto Teresa, Hélida Ferreira da Cunha, Flávia Pereira Lima, João Carlos Nabout

**Affiliations:** 1 Campus de Ciências Exatas e Tecnológicas (CCET), Universidade Estadual de Goiás, Anápolis, Goiás, Brazil; 2 Centro de Ensino e Pesquisa Aplicada à Educação, Campus Samambaia, Universidade Federal de Goiás, Goiânia, Goiás, Brazil; Indiana State University, UNITED STATES

## Abstract

The ability of high school students to know endangered species can vary among species (e.g., large body size can influence people’s interest) or among municipalities (e.g., more contact with biodiversity can influence people’s interest). Thus, in the present paper, we evaluated high school students’ knowledge about the endangered and non-endangered mammalian species of the Brazilian Cerrado. We tested whether the recognition of the endangered and non-endangered species varied in a cross-species analysis (twelve total species) according to species characteristics, such as body size, popularity, endangered status and the length of time of inclusion on the endangered species list. Moreover, we tested whether the recognition of the endangered mammal species varied between municipalities (spatial analysis). We interviewed 366 students in their first year of high school in 21 schools (one in each municipality). Our results indicated that the proportion of correctly identified endangered species varied according to species (cross-species). The endangered species that were most often correctly identified were *Myrmecophaga tridactyla (*known by its popular name, Tamanduá-bandeira, in Brazil), *Priodontes maximus (*Tatu canastra*)* and *Panthera onca* (onça-pintada), with more than 80% correct answers. Thus, students tended to recognize the more popular species and the endangered species more than the non-endangered species. The analysis of student knowledge according to municipality demonstrated that the students’ ability to recognize endangered species followed a spatial pattern. Finally, the cross-species and spatial variation patterns detected in the present study indicated the importance of formal education in increasing high school students’ knowledge about endangered species and suggested that education should also promote less well-known species, species with smaller body sizes, and other groups of vertebrates and invertebrates and consider local and regional biodiversity whenever possible.

## Introduction

The high rate of deforestation and the conversion of native areas into agricultural land and pastures results in an ongoing decrease in biodiversity and a loss of ecosystem services [1]. This entire process has reached a global scale and is now identified as the sixth mass extinction, the first to stem from anthropogenic causes [2, 3]. The human population perceives the impacts of environmental changes but does not effectively engage in actions that promote sustainability [4]. The main cause could be the lack of environmental literacy, which subsidizes decision making [5]. There is an urgent need to understand people’s environmental perception and knowledge to engage stakeholders in effective conservation actions [6, 7, 8]. Human social aspects become a key element in the conservation of species and ecosystems [9, 10].

Any strategy designed to increase local population participation in conservation strategies must begin with a clear comprehension of how native biodiversity and the threats to that biodiversity are perceived. For instance, the naturalization of fish species may alter the perception of the local population regarding invasive species and genuine threats to local biodiversity [11]. Therefore, it is important to assess the initial knowledge of the population and their interest in biodiversity [12]. Common indicators of general public interest are well-recognized species traits, such as a larger body size, a more conspicuous color and a closer similarity to humans [13, 14]. The ecological status of the species (e.g., rarity, degree of threat, native status) also influences people’s interest. More familiar species or those with potential economic uses tend to be preferred by people [15]. These factors do not act in isolation, and often the most preferred species are those with a combination of the abovementioned characteristics; species with these characteristics are called charismatic species [15, 16]. These species appear in animated films and television shows and may contribute to the better recognition of exotic species than native species [17]. The knowledge on biodiversity topics also depends on spatial socioeconomic and environmental factors, such as access to formal and non-formal education, as well as contact and types of experience with nature [18, 19]. For example, social groups with greater access to environmental information (e.g., Internet access and direct contact with species) tend to have more knowledge about species [20, 21]. These variations may occur at large spatial scales [15] but also at smaller scales, such as between municipalities in the same region [19]. Mammalian species are very attractive to the general public, far more so than fish, amphibians and reptiles [12, 16, 22]. Among mammals, the charisma of a species arouses more interest than their endangered status [23]. This is problematic, since the most endangered species need further support, so it is important for the public to know more about these species to influence their attitudes towards conservation-related problems, including the willingness to finance conservation or to accept landscape management strategies [18, 24].

Here, we evaluated high school students’ knowledge about the endangered and non-endangered macrofaunal species of the Brazilian Cerrado. To accomplish this, we tested whether the knowledge of endangered and non-endangered species varied according to species characteristics, such as body size, popularity and time of inclusion on the endangered species list. Our cross-species prediction was that the rate of correct answers by students on species endangered status (endangered or non-endangered) would be greater for endangered, popular, and larger bodied species and for those that had been on the official list of endangered species for a longer time [25, 26]. In addition, we tested whether the knowledge of endangered mammalian species varied between municipalities (spatial analysis). We evaluated whether geographic distance, economic factors, the environmental characteristics of the municipalities, self-reported student recognition (based on a questionnaire regarding each individual’s knowledge of nature) and school characteristics could predict the variation in the students’ performance in recognizing the endangered species. Thus, our spatial-pattern prediction was that students from similar municipalities (environmentally, economically, and educationally) would tend to have similar knowledge of the endangered mammalian species.

## Materials and methods

### Study area

Sampling was carried out in the state of Goiás (Central Brazil), one of the federative units of Brazil, which is part of the Cerrado biome [27]. The Cerrado is a biodiversity hotspot and consists of a mosaic of phytophysiognomies, with high levels of endemism and severely threatened biodiversity [28, 29]. The Cerrado is the second largest biome in Brazil, extending from 2° to 24° south latitude and from 42° to 59° west longitude, and has a seasonal tropical climate [30]. The Cerrado has 5% of all species in the world and 30% of the species in Brazil [31]. More than 50% of the biome has already been deforested, and only 3% is in integral conservation units [32]. One in four endangered species in Brazil is in the Cerrado [33]. A total of 21 municipalities were selected in the state of Goiás (S1 File and S1 Table). In each municipality, one high school was chosen, and the students from that high school were evaluated. We selected municipalities along a gradient of urbanization. For example, municipalities were selected with a demographic density gradient ranging from 2.36 people/km^2^ (Vila Propício) to 1776.74 people/km^2^ (Goiânia).

### Sampling design

We interviewed 366 students in their first year of high school in 21 schools (one from each municipality). Twenty students in their first year of high school were randomly selected from each school. The names of all the students in a class were added in one box, and twenty students were randomly selected. The pilot was performed with 30 students in February 2016 in one school, Anápolis, in Goiás. The pilot data were not included in the statistical tests of the present paper. The sampling occurred between February and April 2016. Consent to participate in this study was obtained in writing from the next of kin, caretakers, or guardians on behalf of the minors/children enrolled in our study. The consent form was sent to students before the study. The study protocol was submitted to and approved by the Ethics Committee of Centro Universitário de Anápolis—UniEvangélica (protocol 41836515.7.0000.5076). Some parents did not return the consent form, and some children were absent on the day of the survey, so the average number of children sampled per school was 17.

The students performed two tasks as follows: a) they answered a questionnaire (objective and subjective questions; S2 File) and b) they classified mammalian species according to their threat of extinction. The purpose of the questionnaire was to measure self-reported knowledge about Cerrado biodiversity and their experiences with biodiversity. In the second task, a packet of figures containing 24 images of mammals from the Cerrado (12 endangered and 12 non-endangered species) was distributed, and the student was instructed to classify each species as either endangered or non-endangered. The student classified one picture at a time. However, when we constructed the dataset (24 pictures), we selected pairs of images of endangered and non-endangered mammals with similar body sizes. This approach is important to reduce classification motivated by some attribute of the photo rather than knowledge about the species. Moreover, the animal’s position within the photo and the landscape were also verified as being similar to prevent any bias in the individual knowledge of the species. The images were obtained from the book “Mammals of Brazil” [34]. We selected species with different body sizes (small and large mammals) and different geographic ranges; however, all species belonged to the Cerrado biome.

### Data analysis

#### Cross-species analysis

The response variable for our cross-species analysis was the percentage of correctly classified endangered and non-endangered species. We tested the prediction based on body size, species popularity, the time since it was classified as endangered, and the endangered category (categorical variables) using a linear model approach. Body size (in centimeters) was obtained from a review of Brazilian mammal species [34], and correspond the average of body size for each species, considering male and female. Popularity was measured using the number of hits on Google after a search with the scientific name of each species in April 2018. In fact, the number of hits on Google can indicate different factors associated with popularity, such as likeability, fear, and disgust; however, this strategy can be used to understand the knowledge of wildlife by society [35]. In addition, a previous study indicated that the use of scientific names in a search of the Internet presented similar results when vernacular names were used (see [36]); thus, in the present paper, we adopted only the scientific name because it is universal and refers to biological organisms. The time since a species was classified as endangered was the number of years that the species has been classified as endangered and included in the Red List of Threatened Species Fauna, published by the Chico Mendes Institute for Biodiversity Conservation (ICMBio). In present paper we used the species classified in follow categories: venerable, endangered and critically endangered. For non-endangered species, the variable time received a value of zero. The endangered category was a dummy variable; non-endangered species were indicated by a 0, and endangered species were indicated by a 1. All these variables were retained in the models because they were not collinear (variance inflation factors < 5; see details in [37] Belsley et al. 2005). All data are available in the supplementary material (S2 Table). We generated 15 linear models that were compared using Akaike’s Information Criterion (AIC) [38]. All variables were standardized (except the endangered dummy variable), and the body size, popularity and number of years classified as endangered were log-transformed (logX + 1). The response variable (percentage of the students who correctly classified the endangered and non-endangered species) was transformed using the arcsine of the square root. The residuals of the regression (using all four predictors) presented a normal distribution (Shapiro-Wilk = 0.96; *p* = 0.48). Akaike’s Information Criterion (AIC) was tested using Spatial Analysis in Macroecology (SAM) software [39].

#### Spatial analysis

Distance matrices were used to investigate the spatial variation in the percentage of the answers correctly recognizing species endangered with extinction. First, the percentage of correct answers of each species for the 21 municipalities was quantified (table with 12 species and 21 municipalities). Second, one matrix of Euclidean distances between municipalities was constructed (*Danswer*), which measured the distance (or similarity) of the percentage of the correct answers between the municipality pairs. The spatial pattern of the correct answers was investigated by relating the *Danswer* matrix to the geographic distance between the municipalities, which was constructed from the Euclidean distances between the geographic coordinates of the municipalities (*Dgeo*). The correlation between the matrices was investigated by the Mantel test with 1000 permutations [40]. In addition, a spatial Mantel correlogram [41] was constructed with five geographical distance classes.

We also performed a Multiple Regression on Distance Matrices (MRM) [42] to evaluate the influence of school, municipality economic status, municipality environmental traits, and student knowledge on the variation in the correct answers of each municipality based on the Euclidian distance between the municipalities (*Danswer*). To construct of the *Danswer* matrix, we estimated the correct answer for each species (column) and each municipality (line). Thus, the *Danswer* matrix was the response variable, and we used the following explanatory matrices: i) the self-reported recognition and contact with biodiversity of the students, ii) the local socio-economic status, iii) the local environmental characteristics, and iv) information about the schools. For the self-reported knowledge and contact with biodiversity of the students, we used the questionnaire to obtain information on the students’ knowledge of biodiversity and endangered fauna. Four quantitative questions (S1 File: q1, q3, q8, and q9) were used to construct a Euclidean distance matrix among the municipalities (*Drecong*). These selected questions agreed with the manual of [43]. For each question, we estimated the average of the answers. This average was used in the statistical test. For the local socioeconomic status, we used the Index of Human Development (IHD), demographic density and per capita Gross Domestic Product (GDP) to construct a Euclidean distance matrix among the municipalities (*Dsoc_econ*). These data were obtained from the Brazilian Institute of Geography and Statistics/IBGE (http://www.cidades.ibge.gov.br/, accessed in February 2016). For the local environmental characteristics, we used the remaining vegetation within the geographic limits of each municipality and the presence of conservation units in the municipality. The remaining vegetation was obtained from geoprocessing maps from the Lapig Laboratory (http://maps.lapig.iesa.ufg.br/lapig.html), and the presence of conservation units was obtained from the Ministry of Environment in the National Registry of Units of Conservation (http://www.mma.gov.br/areas-protegidas/cadastro-nacional-de-ucs) (both sites were accessed in November 2017). We used these variables to construct a Euclidean distance matrix among the municipalities (*Denv*). For information about the schools, we used one of the most commonly used indicators to assess the educational effectiveness of each school in the particular municipalities. This indicator is an index, namely, the Índice de Desenvolvimento da Educação Básica (Basic Education Development Index)–IDEB, organized by the Brazilian government (*Instituto Nacional de Estudos e Pesquisas Educacionais Anísio Teixeira/INEP*, http://ideb.inep.gov.br/resultado/). The IDEB is calculated every two years based on student promotion rates collected by the School Census and is on the averages in reading and mathematics of the two evaluations carried out by the *Instituto Nacional de Estudos e Pesquisas Educacionais Anísio Teixeira—INEP* (National Institute for Educational Studies and Research), the *Prova Brasil*, and the *Sistema de Avaliação da Educação Básica—SAEB* (Basic Education Evaluation System) [44]. We used this index to construct the Euclidean distance matrix among the municipalities (*Dschool*). All data used to perform the matrices are available in the supplementary material (S3 Table).

The data (matrices) were transformed using the arcsine of the square root for the percentage of the data and logX + 1 transformed for the other variables. A multiple regression on the distance matrices (MRM) was performed in R software (https://www.r-project.org/) using the packages asbio [45], vegan [46] and ecodist [42].

## Results

For high school students, the main source of information about biodiversity in the Cerrado was school (78% of the students), followed by television (76%) and the Internet (59%). Moreover, the main threats to the Cerrado biome perceived by the students were deforestation (62%), burning (41%), hunting (24%) and pollution (12%). The other threats to Cerrado biodiversity (e.g., agriculture, urbanization and siltation) totaled 32%.

**Fig 1:**
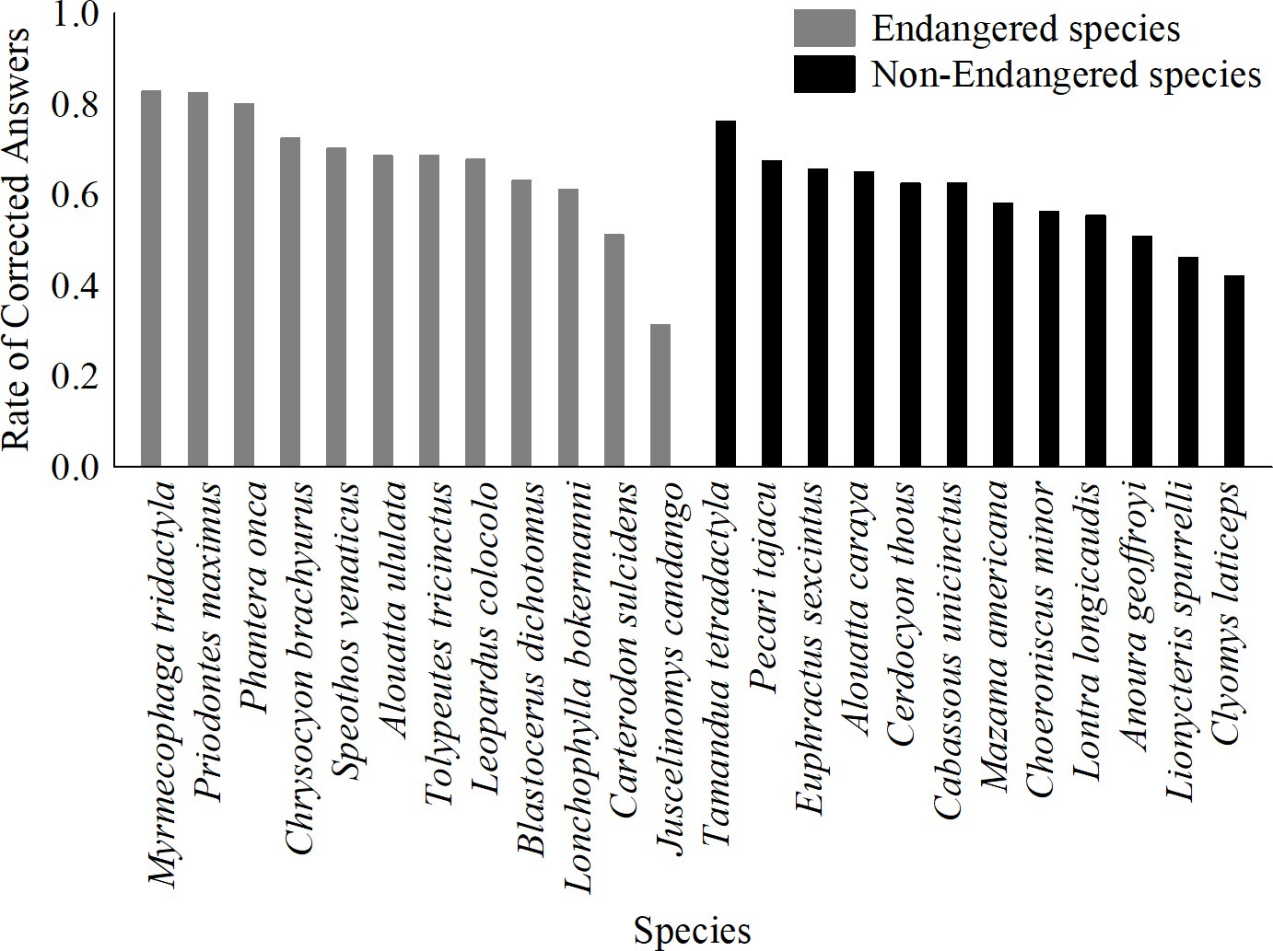
The proportion of student answers correctly identifying the endangered species in 21 public high schools.

The proportion of the student answers correctly identifying the endangered species varied according to species (cross-species). The endangered species that received the highest percentages of correct answers were *Myrmecophaga tridactyla*, *Priodontes maximus* and *Panthera onca*, with more than 80% correct (Fig 1). The species with the lowest proportion of correct answers was *Juscelinomys candango*, with 31.4%. Otherwise, the non-endangered species that received the highest rate of correct answers was *Tamandua tetradactyla*, with more than 76% correct, and the non-endangered species with the lowest rate of correct answers was *Clyomys laticeps*, with 42.7% (Fig 1).

Fifteen different models explaining the influence of body size, popularity, time and the endangered status of a species on the number of correct answers according to species were generated (Table 1). The best model was formed by two variables (popularity and endangered status) and explained 61.5% of the variation in the number of correct answers among the endangered and non-endangered species (Table 1); moreover, three models had ΔAIC scores lower than 2, and the variables popularity, endangered and time were present in these models. Thus, body size was not present in the better models. Popularity and endangered status were variables that were more important in explaining the variation in the rate of correct answers by the students (Table 2). Students tended to correctly assign the popular and endangered species as endangered more than the less popular and non-endangered species. On the other hand, the time since a species had been on the Red List and body size had low explanatory power (Table 2).

**Table 1:** Models generated to explain the variation in the number of correct answers according to species. Each model shows the determinant coefficient (R^2^), the Akaike’s Information Criterion (AIC) score, the difference in the AIC score from that of the best model (ΔAIC), and the AIC weights (AIC Wi). The variables used were body size (BS), popularity (Pop), length of time of inclusion on the Red List (Time), and endangered status (End). See details in the Materials and Methods.

Models	R^2^	AIC	Delta AIC	AIC wi
Pop, End	0.615	-42.527	0	0.315
Pop, Time, End	0.649	-41.531	0.996	0.191
Pop	0.544	-41.41	1.117	0.18
Pop, Time	0.578	-40.369	2.158	0.107
BS, Pop, End	0.617	-39.421	3.106	0.067
BS,Pop	0.558	-39.235	3.292	0.061
BS, Pop, Time, End	0.649	-37.96	4.567	0.032
BS, Pop, Time	0.584	-37.483	5.044	0.025
BS	0.426	-35.889	6.638	0.011
BS, End	0.456	-34.264	8.263	0.005
BS, Time	0.441	-33.619	8.908	0.004
BS, Time, End	0.468	-31.562	10.964	0.001
End	0.101	-25.104	17.422	<0.001
Time	0.072	-24.351	18.176	<0.001
Time, End	0.108	-22.386	20.141	<0.001

**Table 2:** The importance and standardized angular coefficient (std. coeff.) of each predictor used in the model selection (see Table 1). The variables used were body size (BS), popularity (Pop), length of time of inclusion on the Red List (Time), and endangered status (End, dummy variables, where 0 indicated a non-endangered species and 1 indicated an endangered species). See details in the Materials and Methods.

Variables	Importance	Std. Coeff
BS	0.206	0.168
Pop	0.978	0.712
Time	0.361	-0.265
End	0.612	0.447

**Fig 2:**
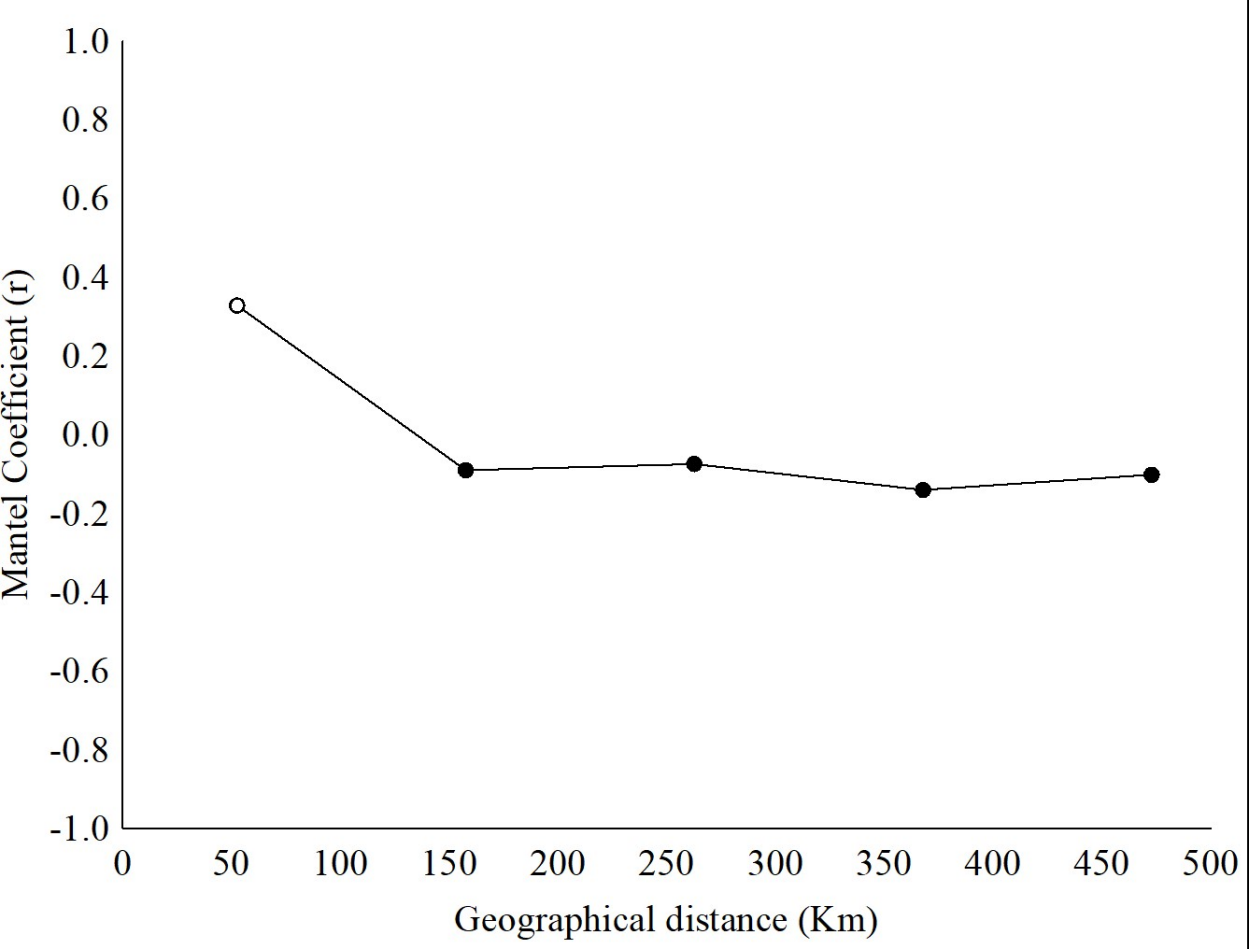
The Mantel correlogram indicated the spatial structure of the percentage of correctly identified endangered species (Danswer) along the geographical distance. The x-axis indicates the geographic distance class (in km), and the y-axis indicates the r of the Mantel test. The open circle indicates significant values of r (*p*<0.005).

Considering that the students were able to recognize the endangered species, we used the endangered data to model the spatial variation of the rate of correct answers among the 21 municipalities. Thus, the spatial analysis indicated a significant spatial pattern within the first distance class. In the other words, geographically close municipalities tended to present similar percentages of correct answers about endangered species, and increasing the geographical distance between the municipalities tended to result in differences in the percentages of correct answers (Fig 2). This spatial pattern was most evident in municipalities up to 53 km away from each other, and municipalities farther apart than this threshold tended to present more distinct results (i.e., different percentages of correct answers).

We used four predictors to explain the spatial variation in the percentages of correct answers (among municipalities), namely, socioeconomic characteristics (indicated by many socioeconomical indices, e.g., the IHD), local environmental characteristics (remaining vegetation and the presence of conservation units), school characteristics (indices to assess educational effectiveness) and self-reported student recognition (quantitative questions in questionnaire). The MRM showed that only “self-reported recognition” explained the variation in the percentage of correct answers (r = 0.11; *p* = 0.04). In other words, municipalities with similar student knowledge (based on questionnaires) had similar percentages of correctly identified endangered species (Table 3).

**Table 3:** The multiple regression on the distance matrices (MRM) indicated the standardized coefficient and P-value of each matrix predictor. The response matrix was the percentage of correct answers among the municipalities (*Danswer*).

Matrices	Standardized coeff	*p*
Local Socio_Economic (*Dsoc_econ*)	-0.02	0.39
Self-reported Recognition (*Drecong*)	0.11	0.04
Local Environmental Characteristics (*Denv*)	-0.01	0.43
School (*Dschool*)	-0.03	0.9

## Discussion

The present paper used two approaches to investigate students’ knowledge about the endangered and non-endangered mammalian of the Cerrado (cross-species) and patterns in spatial variation. Our results showed substantial variation in the percentage of correct answers according to species and localities. These results are in accord with our expectations that the knowledge of a species varies according to the attributes of the species and the previous experiences of the students with biodiversity. The rate of correct answers of a species status (endangered and non-endangered) was higher for the endangered species, suggesting that students were able to recognize the endangered species from the non-endangered species. Moreover, the students’ performance in the recognition of the endangered species is explained by species popularity, which partially supports our species-based hypothesis. However, the spatial variation in the percentage of correct answers about endangered species among the municipalities was explained by the self-reported recognition of the students rather than socioeconomic status or school characteristics. These results partially support our spatial pattern hypothesis.

Our results indicated that the students’ ability to recognize the endangered status of a species is based on the endangered status of the species itself and on species popularity. Therefore, popularity seems to improve the capacity of recognition of an endangered species, highlighting the importance for scientists to create documents (sites and books among other resources) to increase the knowledge of wildlife by society [35]. In fact, the endangered species had more pages on the Internet than the non-endangered species (endangered = 169120.8 pages; non-endangered = 34549.14). However, depending only on popularity leads to a valorization of the charismatic species [23]. Therefore, educational conservation projects should engage students in active learning, promote outdoor learning practices, incorporate real life elements such as documentaries and newspapers to broaden learning about the environment and promote changes in knowledge and attitudes [47, 48, 49]. In addition, according to our results, it is possible that the most popular mammals are more frequently cited as examples in biology classes, since this group of vertebrates is the most frequently represented in photographs in Brazilian textbooks [50].

In the present study, body size was less important for explaining the variation in the number of correct answers among the endangered and non-endangered species. It can be illustrated by the low rate of correct answers of some large-bodied species, such as brocket deer (*Mazama americana*) and the largest South American deer (*Blastocerus dichotomus*), which had lower rates of correct classification in relation to their endangered status. Furthermore, students correctly classified some small-bodied species, such as the endangered armadillo (*Tolypeutes tricinctus*). These examples are contrary to the idea that larger-bodied species arouse greater public interest [51, 52, 53], which, in turn, could be related to having more information and the capacity of recognition by the students. However, it is worth mentioning that despite comparatively not including BS in the best models, their predictive power is considerable. In fact, the model composed solely of BS explains 42% of the variation in the responses by the students.

The analysis of the spatial pattern found in this study indicates that the students’ ability to recognize the endangered species follows a spatial pattern, but only the knowledge of the students was able to explain this spatial variation. This variable represents the students’ self-reported knowledge about biodiversity, access to information (school, Internet, magazines, and television) and direct contact with nature. In fact, personal experience in natural environments increases knowledge about biodiversity and environmental awareness, which affects conservation-friendly behavior [19, 54]. For example, in a study developed in Brazil, students from schools that were geographically more distant from a conservation unit (Canela National Forest) were less sensitive to environmental issues. Therefore, the experience of students with a conservation unit causes them to perceive the environment in which they live in a new way. Non-formal contact was also shown to be effective in the "Nature on the Way to School" project, which highlighted local species to Swiss students on the way to their schools, changing their environmental knowledge about exotic and native species [55]. In the present paper, the rate of correct answers about the endangered species was not affected by urbanization and the proximity of nature (see predictor local environmental characteristics), and this result reinforces the importance of formal and non-formal education in the perceptions of the students about biodiversity. In other words, education promotes students’ perceptions of endangered species regardless of the level of urbanization and preservation of the municipality in which the students live. Therefore, didactic materials and visits to parks and zoos among other resources can promote an increase in the environmental perception of the students.

The economic and school factors (IDEB score) had no significant power to explain the frequency of correct answers given by the students. These results suggest that students living in municipalities with different economic conditions and levels of school performance do not differ in their frequency of correct answers. In fact, the IDEB score is highly correlated with the socioeconomic conditions of the students and the municipalities, and it does not necessarily capture specific student educational skills such as mathematics and reading [44]. Specific pedagogical practices adopted by schools or municipalities, different levels of prior biological knowledge and knowledge of students are examples of factors that may not be captured by the official assessment. In addition, other variables that also present spatial patterns could explain the spatial patterns of the correct student answers. For example, in nearby municipalities (less than 53 km), there is a constant exchange between students and teachers, regular continuing education courses and university extension activities, which would promote greater similarities between the schools.

Finally, the questionnaire information and the students’ recognition of the endangered species indicated the positive attitudes of the students towards conservation of the Cerrado. Therefore, the dissemination of science, the production of materials on biodiversity and formal education has contributed to raising awareness among the students. Moreover, the present study adds new information to the scientific literature on society’s environmental knowledge. Considering our main results (variation between species and between municipalities), we recommend that greater efforts be directed towards scientific communication, environmental education activities and teacher training to increase society’s knowledge about native species that are endangered with extinction. According to our results, education should also promote non-popular and smaller species, including other groups of vertebrates and invertebrates, while considering local and regional biodiversity whenever possible. The importance of this education is highlighted by the fact that people’s engagement with the conservation of a species is directly related to their understanding of nature [10], which is often guided by emotions and not associated with the vulnerability of the species [23].

## Supporting information

S1 File Municipalities used in the present study. In each municipality, one high school was selected.
(DOCX)

S2 File Questionnaire given to the students in the 21 municipalities. (DOCX)

S1 Table Municipality and state school (S.S.) visited in the present paper. (DOCX)

S2 Table Information about the twenty-four species used in the present paper. The category of threatened is according to the Red List of ICMBIO (see details in the Methods section). The % of correct answers indicates the percentage of correct answers considering all municipalities. Body size (cm), time (year), popularity (number of pages where the species appeared on a Google search in April 2018).
(DOCX)

S3 Table All data used for the spatial analysis. Geographic coordinates, names of the municipalities, percent of correct answers by species (columns D to O), Index of Human Development (IHD), demographic density and per capita gross domestic product (GDP), average for each question in the questionnaire (columns R to Z), Índice de Desenvolvimento da Educação Básica (IDEB by school, and by municipalities), percent of remaining vegetation and presence of conservation unity (where 1 indicates presence and zero is the absence of conservation unity).
(XLSX)
