# Supplementary material for: High school students’ knowledge of endangered fauna in the Brazilian Cerrado: A cross-species and spatial analysis
Source: PLoS One. 2019 Apr 25;14(4):e0215959. doi: 10.1371/journal.pone.0215959 (PMC6483199; doi:10.1371/journal.pone.0215959)

**S1 File. Municipalities used in the present study.** In each municipality, one high school was selected.


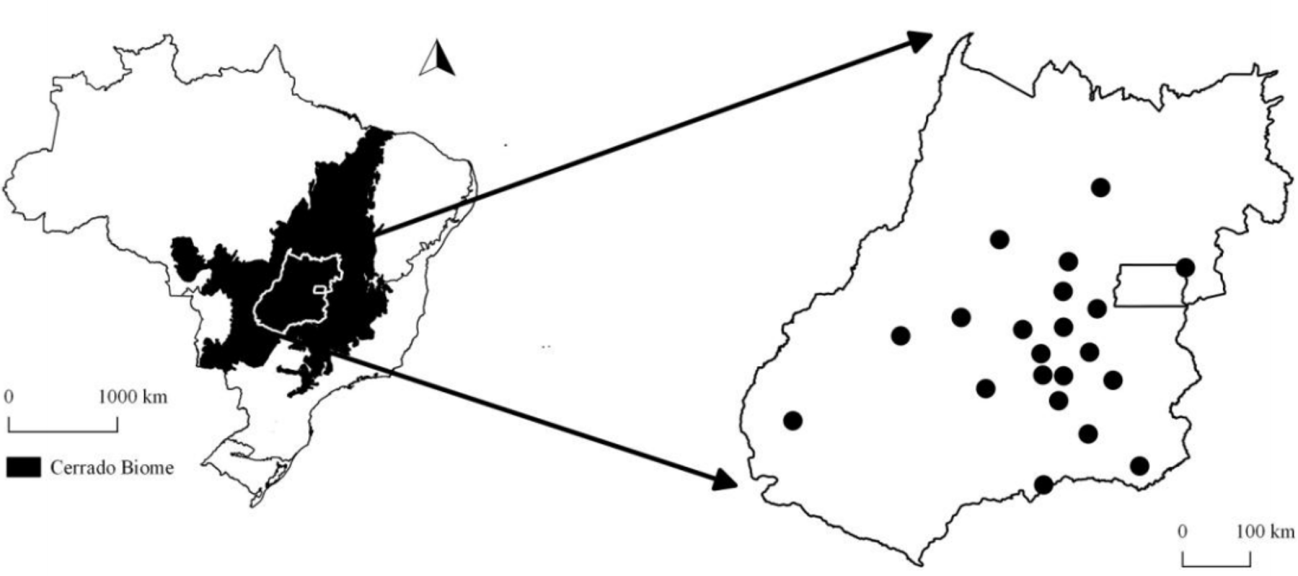

Supplement: S1 File — In each municipality, one high school was selected. (DOCX) [file pone.0215959.s001.docx]
