# Supplementary material for: High school students’ knowledge of endangered fauna in the Brazilian Cerrado: A cross-species and spatial analysis
Source: PLoS One. 2019 Apr 25;14(4):e0215959. doi: 10.1371/journal.pone.0215959 (PMC6483199; doi:10.1371/journal.pone.0215959)
Supplement: S2 File — (DOCX) [file pone.0215959.s002.docx]

**S2 File. Questionnaire Applied for Students into 21 municipalities.**

Biodiversity of the Cerrado and Threatened Species

Basic Guidelines for Filling:

1 - You should provide the information requested in the first part but should not indentify yourself by name.

2 - Mark “X” on the answer below that better reflects your opinion.

Personal Information

Age: ___________ Gender: (   ) Male (   ) Female

What municipality does you live: ___________________________________________

**Questionnaire**

q1 - What knowledge about diversity of species in the Cerrado biome do you have? Consider 0 as none knowledge or 10 as high knowledge.

( ) 0 ( ) 6

( ) 1 ( ) 7

( ) 2 ( ) 8

( ) 3 ( ) 9

( ) 4 ( ) 10

( ) 5

q2 - How did you hear about biodiversity? You can choose more than one option below.

(   ) I never heard (   ) Internet

(   ) Talking with friends (   ) Magazines

(   ) School ( ) Newspapers

( ) TV

q3 - How many times have you searched for biodiversity of Cerrado biome in the lasts months (TV, Internet, Newpapers, Magazines)? Indicate the number of times do you remember: __________________

q4 - How often have you searched about threatened species of the Cerrado biome at last month (TV, Internet, newspapers, Magazines)? Indicate the number of times do you remember: __________________

q5 - Do you know some kind of threat attributed from humans action to Cerrado species? (   ) Yes (   ) No

q6 - If Yes, which one? __________________________________________________

q7 - Did you already hear about the list of threatened species of extinction from Brazil?

( ) Yes ( ) No

q8 - How many times did you already go in the Zoo of your city or in any other city? Indicate the number of times do you remember: ____________________

q9 - How often (days) do you go to the nature regions (farm, fishery, environmental parks) per month? Indicate the number of times do you remember: ________________
