# Supplementary material for: High school students’ knowledge of endangered fauna in the Brazilian Cerrado: A cross-species and spatial analysis
Source: PLoS One. 2019 Apr 25;14(4):e0215959. doi: 10.1371/journal.pone.0215959 (PMC6483199; doi:10.1371/journal.pone.0215959)
Supplement: S1 Table — (DOCX) [file pone.0215959.s003.docx]

**S1 Table.** Municipality and State School (S.S.) visited in present paper.

| **Municipality** | **Name of School** |
| --- | --- |
| ALEXANIA | S.S. 31 DE MARCO |
| ANAPOLIS | S.S. PROFESSOR HELI ALVES FERREIRA |
| BELA VISTA DE GOIAS | S.S. PEDRO VIEIRA JANUÁRIO |
| CALDAS NOVAS | S.S. CALDAS NOVAS |
| CATALAO | S.S. POLIVALENTE DOUTOR THARSIS CAMPOS |
| INDIARA | S.S. DE INDIARA |
| ORIZONA | S.S. MARIA BENEDITA VELOZO |
| PIRACANJUBA | S.S. RUY BRASIL CAVALCANTE |
| PIRENOPOLIS | S.S. COMENDADOR CHRISTOVAM DE OLIVEIRA |
| SILVANIA | S.S. PROF. JOSÉ PASCHOAL DA SILVA |
| VILA PROPICIO | S.S. DOM PEDRO II |
| FORMOSA | S.S. DR. JOSÉ BALDUÍNO DE SOUZA DÉCIO |
| GOIANIA | S.S. POLIVALENTE PROFESSOR GOIANY PRATES |
| HIDROLINA | S.S. ALFREDO NASSER |
| INHUMAS | S.S. RUY BARBOSA |
| IPORA | S.S. OZÓRIO RAIMUNDO DE LIMA |
| ITUMBIARA | S.S. POLIVALENTE DOUTOR MENEZES JUNIOR |
| MINEIROS | S.S. POLIVALENTE ANTÔNIO CARLOS PANIAGO |
| NIQUELANDIA | S.S. PAULO FRANCISCO DA SILVA |
| RUBIATABA | S.S. PEDRO ALVES DE MOURA |
| SANCLERLÂNDIA | S.S. DEPUTADO JOSE ALVES DE ASSIS |
