# Supplementary material for: High school students’ knowledge of endangered fauna in the Brazilian Cerrado: A cross-species and spatial analysis
Source: PLoS One. 2019 Apr 25;14(4):e0215959. doi: 10.1371/journal.pone.0215959 (PMC6483199; doi:10.1371/journal.pone.0215959)
Supplement: S2 Table — The category of threatened is according to the Red List of ICMBIO (see details in the Methods section). The % of correct answers indicates the percentage of correct answers considering all municipalities. Body size (cm), time (year), popularity (number of pages where the species appeared on a Google search in April 2018). (DOCX) [file pone.0215959.s004.docx]

**S2 Table.** **Information about twenty four species used in present paper.** The category of threatened is according red list of ICMBIO (see details in methods). The % of Correct answers indicate the percentage of correct answers considering all municipalities. The body size (Cm), Time (year), Popularity (number of pages where the species appeared on a Google search in April 2018).

| Category | Species | Order | Category of Threatened | Geographic Distribution | % of Corrected answer | Body Size | Popularity | Time |
| --- | --- | --- | --- | --- | --- | --- | --- | --- |
| Endangered | *Alouatta ululate* | Primates | Endangered | Caatinga, Cerrado and magroves | 68.58 | 52.5 | 3640 | 11 |
|  | *Blastocerus dichotomus* | Artiodacyla | Vulnerable | Pantanal and Cerrado | 63.11 | 172 | 44200 | 25 |
|  | *Carterodon sulcidens* | Rodentia |  | Cerrado | 51.09 | 17.75 | 3060 | 11 |
|  | *Chrysocyon brachyurus* | Carnivora | Vulnerable | Cerrado and Pampa | 72.40 | 105 | 195000 | 25 |
|  | *Juscelinomys candango* | Rodentia | Critically Endangered | Cerrado | 31.42 | 14.15 | 1720 | 25 |
|  | *Leopardus colocolo* | Carnivora | Vulnerable | Cerrado, Pantanal and Pampa | 67.76 | 80 | 36700 | 1 |
|  | *Lonchophylla bokermanni* | Chiroptera | Vulnerable | Cerrado | 61.20 | 4.85 | 4130 | 11 |
|  | *Myrmecophaga tridactyla* | Pilosa | Vulnerable | Neotropic | 82.79 | 110 | 146000 | 11 |
|  | *Phantera onca* | Carnivora | Vulnerable | Neotropic | 80.05 | 197.7 | 1460000 | 25 |
|  | *Priodontes maximus* | Cingulata | Vulnerable | Neotropic | 82.51 | 87.5 | 54300 | 11 |
|  | *Speothos venaticus* | Carnivora | Vulnerable | Neotropic | 70.22 | 70 | 57200 | 25 |
|  | *Tolypeutes tricinctus* | Carnivora | Vulnerable | Caatinga and Cerrado | 68.58 | 30 | 23500 | 25 |
| Non-Endangered | *Alouatta caraya* | Primates | non-endangered | Cerrado, Pantanal and Amazon | 65.03 | 52.5 | 45500 | 0 |
|  | *Anoura geoffroyi* | Chiroptera | non-endangered | Cerrado and Atlantic | 50.82 | 7 | 13900 | 0 |
|  | *Cabassous unicinctus* | Cingulata | non-endangered | Cerrado, Amazon, Pantanal, Atlantic | 62.57 | 39.6 | 12700 | 0 |
|  | *Cerdocyon thous* | Carnivora | non-endangered | All brazilian biome | 62.57 | 65 | 76000 | 0 |
|  | *Choeroniscus minor* | Chiroptera | non-endangered | Cerrado, Amazon | 56.28 | 5.3 | 5540 | 0 |
|  | *Clyomys laticeps* | Rodentia | non-endangered | Cerrado | 42.08 | 17.7 | 6140 | 0 |
|  | *Euphractus sexcintus* | Cingulata | non-endangered | Cerrado, Amazon, Pantanal, Pampa | 65.57 | 40 | 31700 | 0 |
|  | *Lionycteris spurrelli* | Chiroptera | non-endangered | Amazon, Cerrado | 46.17 | 5 | 5110 | 0 |
|  | *Lontra longicaudis* | Carnivora | non-endangered | All brazilian biome | 55.46 | 66.5 | 47800 | 0 |
|  | *Mazama americana* | Cetartiodactyla | non-endangered | Cerrado, Amazon, Pantanal, Atlantic | 58.20 | 133.5 | 43900 | 0 |
|  | *Pecari tajacu* | Artiodacyla | non-endangered | Cerrado, Pantanal, Atlantic, Amazon | 67.49 | 90 | 69700 | 0 |
|  | *Tamandua tetradactyla* | Pilosa | non-endangered | All brazilian biome | 76.23 | 62 | 56600 | 0 |
